# Proteomic response of A549 lung cancer cell line to protein-polysaccharide complex Venetin-1 isolated from earthworm coelomic fluid

**DOI:** 10.3389/fmolb.2023.1128320

**Published:** 2023-06-08

**Authors:** Paulina Czaplewska, Aleksandra Bogucka, Katarzyna Macur, Magda Rybicka, Michał Rychłowski, Marta J. Fiołka

**Affiliations:** ^1^ Intercollegiate Faculty of Biotechnology, The University of Gdansk, Gdańsk, Poland; ^2^ Institute of Biochemistry, Justus Liebig University of Giessen, Giessen, Germany; ^3^ Department of Immunobiology, Institute of Biological Sciences, Maria Curie-Skłodowska University, Lublin, Poland

**Keywords:** protein-polysaccharide complex, coelomic fluid, earthworms, proteomics, swath

## Abstract

Earthworms’ celomic fluid has long attracted scientists’ interest due to their toxic properties. It has been shown that the elimination of coelomic fluid cytotoxicity to normal human cells was crucial for the generation of the non-toxic Venetin-1 protein-polysaccharide complex, which exhibits selective activity against *Candida albicans* cells as well as A549 non-small cell lung cancer cells. To find the molecular mechanisms behind the anti-cancer properties of the preparation, this research investigated the proteome response of A549 cells to the presence of Venetin-1. The sequential window acquisition of all theoretical mass spectra (SWATH-MS) methodology was used for the analysis, which allows for a relative quantitative analysis to be carried out without radiolabelling. The results showed that the formulation did not induce significant proteome responses in normal BEAS-2B cells. In the case of the tumour line, 31 proteins were up regulated, and 18 proteins down regulated. Proteins with increased expression in neoplastic cells are mainly associated with the mitochondrion, membrane transport and the endoplasmic reticulum. In the case of altered proteins, Venetin-1 interferes with proteins that stabilise the structures, i.e., keratin, glycolysis/gluconeogenesis and metabolic processes.

## Introduction

The human body functions efficiently thanks to the synchronised work of many vital organs, such as the heart, brain, lungs, and liver. However, during our lifetime, each of them is exposed to numerous diseases and cell changes like, for example, cancer. It affects the entire age cross-section of society, and despite numerous studies, there are still no effective therapies for all of them. That is why it is essential to constantly search for and research compounds, especially those of natural origin, that show anti-cancer properties or allow for the strengthening or targeting of modern therapies.

One of the critical cancers is lung cancer, represented mainly by non-small cell lung cancer (NSCLC), including lung adenocarcinoma (Molina et al., 2008; Yu et al., 2020). Often, its appearance is associated with the patient’s exposure to toxic substances, mainly tobacco smoke (both passive and active smoking), and the inhalation of toxins in the air (Yue et al., 2017; Schuller, 2019). The last environmental factor is significant today, and the main factors in the air negatively affecting the lungs are sulfur oxides, nitrogen oxides or dust with a diameter of less than 2.5 µm (Hamra et al., 2014; Hamra et al., 2015). Malignant lung changes may also appear regardless of the factors mentioned, which do not change the symptoms or the course of the disease itself. In all cases, the prognosis for patients is cautious or poor (Woodard et al., 2016).

Among the many natural compounds with anti-cancer potentials, such as curcumin (Xiang et al., 2019), concerning NSCLC, preparations of earthworms origin attract attention. They have been used in therapy in Asia since ancient times as powders, liquid extracts of dried earthworms or pastes (Shen, 2010; Cooper et al., 2012; Sun, 2015; Wang et al., 2021). Currently, research has confirmed such properties of the coelomic fluid (CF) naturally filling the body cavity of the earthworm (Fiołka et al., 2021). CF is a multi-component preparation containing metabolites, proteins, enzymes, peptides, and polysaccharides. The coelomic fluid of the earthworm exhibits many types of biological activity, such as antimicrobial, proteolytic, hemolytic, hemagglutinating, antifungal, and antitumor activity. In the case of coelomic fluid, anti-cancer properties against cancer cell lines were confirmed for cells like HeLa, A549, Pa17, PC-12, or Hep-2 (Milochau et al., 1997; Yanqin et al., 2007; Fiołka et al., 2019a; Czerwonka et al., 2020; Fiołka et al., 2021). The problem, however, is the toxicity of the fluid to the vertebrate cells (Kobayashi et al., 2001). It turns out, as presented by Fiołka and colleagues, that the processing of the fluid associated with heating for a short time at 70°C changes the toxic properties of the raw fluid (Fiołka et al., 2019b). It becomes non-toxic to normal cells (does not cause red blood cell hemolysis) and can still affect cancer cells (Rybicka et al., 2022). Additionally, it exhibits documented antifungal activity and can fight *Candida albicans* (Fiołka et al., 2021). Analysis of the composition of the fraction obtained by heating dialysis, initially named by the authors as Venetin-1 (formerly protein-polysaccharide fraction), contains proteins and sugars. The main protein components are lysenin, lysenate and lysenin-related protein 2 (LRP2) proteins (Milochau et al., 1997; Kobayashi et al., 2001; Yanqin et al., 2007; Fiołka et al., 2019a; Fiołka et al., 2019b; Czerwonka et al., 2020; Fiołka et al., 2021). These proteins, belonging to the group of pore toxins, are naturally associated with the fluid’s toxicity and the earthworm’s protection against environmental pathogens by creating pores (Shakor et al., 2003; Sukumwang and Umezawa, 2013). The binding of lysenin to the nonamer allows for structural reorganisation and penetration of the cell membrane through the created pore (Shrestha et al., 2020). Therefore, further research using Venetin-1 and determining its full potential as the molecular mechanism responsible for its operation is needed. Currently, mass spectrometry (MS) is an excellent support for the methods of molecular biology and biotechnology in the study of mechanisms. The development of quantitative methods in MS based on both isotope labelling of preparations (SILAC, TMT, iTRAQ) and methods without labelling (by peak intensity, by spectral count, SWATH-MS) makes it possible to study the responses of various types of proteomes to anti-cancer preparations (Zhu et al., 2009; Sap and Demmers, 2012; Lindemann et al., 2017). Sequential Window Acquisition of All Theoretical Mass Spectra (SWATH-MS) is nowadays a powerful platform allowing for relative estimation of protein levels (Schubert et al., 2015; Collins et al., 2017; Ludwig et al., 2018; Janacova et al., 2020; Lewandowska et al., 2021). The first step in SWATH MS analysis is to prepare a library based on the information dependent acquisition (IDA) method and then record the fragmentation spectra of all samples in a data independent acquisition (DIA) format. At this stage, all precursor ions are fragmented in fixed or variable isolation windows, which covers virtually the entire m/z range. The last step is retrospective interrogation of the peptides of interest using previously registered spectral library. The digital record of proteome changes obtained can be subjected to functional analysis. More importantly, it can be analysed many times at longer intervals without needing to re-register the spectra, deposited and made available among scientific groups from around the world.

In the present studies, the SWATH MS strategy was used to determine the proteome response of human lung cancer cell line A549 to the presence of the earthworm preparation, Venetin-1. The studies showed no significant changes in the proteome of BEAS-2B control cells cultured in the presence of Venetin-1, while the tumour line showed changes in the mitochondrion and the protein levels directly connected with the work of the endoplasmic reticulum. It is the first step in understanding the anti-cancer properties of this natural preparation concerning lung cancer.

## Materials and methods

### Earthworms

*Dendrobaena veneta* (Supplementary Figure S1), belonging to invertebrates, is an ethically uncontroversial organism and does not require obtaining permits to conduct research. The earthworms for research were kept in the laboratory culture of the Department of Immunobiology of Maria Curie-Skłodowska University (Lublin, Poland). The model organisms were grown in containers filled with compost soil and fed with boiled vegetables and pure cellulose necessary for cocoon formation. The breeding of earthworms has been conducted in complete darkness at a temperature of about 20–22°C and a humidity of about 70%. Only adult specimens were selected for the study.

### Obtaining Venetin-1 from coelomic fluid

The earthworms were subjected to intestinal cleansing by being kept on moist lignin for 24 h. Mild electrostimulation with a voltage of 4.5 V lasting 30 s was used to collect the celomic fluid. The celomic fluid was collected in a physiological fluid (NaCl 0.9%) in a glass vessel. The coelomocytes were then separated by centrifugation at 4,629 *g* for 10 min. The supernatant was filtered through 0.22 µm Millipore filters and then dialysed in water at 4°C for 24 h. After dialysis, the preparation was freeze-dried and frozen at −20°C. The Bradford method (Bio-Rad) determined the protein concentration in the preparation’s water solution.

### Cell Viability and Apoptosis

The human bronchial epithelial cells (BEAS-2B) and lung adenocarcinoma epithelial cell line (A549 cells) were cultured in RPMI 1640 medium (Gibco, Invitrogen, United States) supplemented with 8% FBS (Gibco, Invitrogen, United States), 5% penicillin/streptomycin (Gibco, Invitrogen, United States). The cytotoxicity effect of Venetin-1 on BEAS-2B and A549 cells was determined by the methylthiazol tetrazolium (MTT) assay. Briefly, cells were seeded in a 96-well plate at a density of 1 × 10^4^ cells/well and allowed to adhere for 24 h. Next, the medium was removed and replaced with 100 μL of fresh medium containing serial concentrations of Venetin-1 (15.62, 31.25, 62.5, 125, 250, and 500 μg/mL). Each 96-well plate contained three controls (cells-only) and three zero-adjustment (medium-only) wells. After 72 h, the culture medium was replaced with a fresh medium, and cells were treated with 20 µL of MTT (Sigma, St. Louis, MO) working solution (5 mg/μL) at 37°C. Four hours later, the solution was removed, and 100 μL of DMSO (Sigma-Aldrich) was added to each well to solubilise the purple formazan crystals. The absorbance was determined after 1 h at 550 nm with a plate reader Victor 1,420 multilabel counter (PerkinElmer, Inc. Waltham, MA United States).

FITC-Annexin V Apoptosis Detection Kit (BDBiosciences, San Jose, CA) measured apoptosis induced by Venetin-1. For cell apoptosis detection, A549 cells were plated on 6-well plates (2 × 10^5^ cells/mL) for 24 h and then treated with 125 μM Venetin-1 for 72 h. Samples were prepared according to the manufacturer’s protocol and analysed on a Guava easyCyte flow cytometer (Merck, Germany).

### Detection of autophagy by acridine orange staining

The A549 and BEAS-2B cells were seeded on 6-well plates (1 × 10^6^ cells/mL) for 24 h and treated (+) or untreated (−) with 125 μg/mL Venetin-1 for 72 h. Subsequently, the cells were stained with 0.1 μg/mL water solution of acridine orange (Invitrogen) for 10 min at 37°C. Specimens were imaged using a confocal laser scanning microscope (Leica SP8X equipped with an incubation chamber for the live analysis) with a 63× oil immersion lens (Leica, Germany). Excitation 498 nm, emission 511nm–549 nm (green), 601nm–719 nm (red).

### Sample preparation

BEAS-2B and A549 cells were seeded onto 6-well microtiter plates at 2 × 10^5^ cells/mL density. After 24 h, the culture medium was replaced with the standard medium for control cells (BEAS-2B-, A549-), and for the treated groups (BEAS-2B+, A549+) it was replaced with a medium containing 125 μg/mL of Venetin-1. The cells were collected, washed twice with PBS (Gibco, Invitrogen, United States), and centrifuged for 5 min at 1200 g to collect the cell pellet.

### Sample digestion protocol

The standard FASP method protocol was used for protein extraction and digestion 32. Two Venetin-1 treated and untreated cell lines were submitted for proteomic analysis (A549 and BEAS-2B). Briefly, cells were lysed by treatment with lysis buffer (1% SDS, 100 mM Tris/HCl pH 8, 50 mM DTT) and then heated for 10 min at 95 °C. After heating, the concentration of the released proteins was measured spectrophotometrically (Multiscan Thermo), and 100 μg of protein was taken for each sample for further digestion. The FASP procedure was performed on a 10 kDa mass cut-off membrane, and the digestion was performed with trypsin (Czaplewska et al., 2021). After digestion and collection the tryptic peptides, the final purification was performed according to the StageTips procedure on the C18 phase, where 10 μg of peptides were taken for purification based on spectrophotometric concentration measurement (Rappsilber et al., 2007).

### Mass spectrometry analysis

Spectrum registration was performed on a TripleTOF 5600+ (Sciex Framingham, MA, United States) mass spectrometer connected to a chromatography system, the Ekspert MicroLC 200 Plus System (Eksigent, Redwood City, CA, United States). All chromatographic separations were performed on the ChromXP C18CL column (3 μm, 120 Å, 150 × 0.3 mm). For each sample, the chromatographic gradient for each MS run was 11%–42.5% B (solvent A 0% aqueous solution, 0.1% formic acid; solvent B 100% acetonitrile, 0.1% formic acid) in 60 min. The whole system was controlled by the SCIEX Analyst TF 1.7.1 software. Measurements for the spectral library were acquired in data-dependent acquisition (DDA) mode. Each cycle of the applied DDA method comprised precursor spectra accumulation in 100 ms in the range of 400–1,200 m/z followed by top 20 precursor’s product ion spectra accumulation in 50 ms in the range of 100–1800 m/z, resulting in a total cycle time of 1.15 s. Formerly fragmented precursor ions were dynamically excluded.

### SWATH-MS and data analysis

The sequential window acquisition of all theoretical mass spectra (SWATH-MS) method was used to quantify proteins. Experiments were performed in a looped product ion mode with the spectrometer set to high sensitivity focus. A set of 25 transmission windows of variable width was constructed using SwathTUNER software based on the equalised frequency of precursor ions and covering the precursor mass range of 400–1,200 m/z. The collision energy for each window was calculated for +2 to +5 charged ions centred upon the window with a spread of five. The SWATH-MS survey scan was acquired in the range covered by constructed windows at the beginning of each cycle, with an accumulation time of 50 ms. Product ion scans were collected in the range of 100–1800 m/z in 40 ms, which resulted in a total cycle time of 1.1 s.

For quantitative analysis of four sample types, a spectral library was created with the group file data processing in PeakView v. 2.2 (SCIEX), with settings described in detail by Lewandowska (Lewandowska et al., 2021). A joint search for library generation included all measurements conducted in DDA mode. For database search, ProteinPilot 4.5 software (Sciex) was used. It is based on the Paragon algorithm against the dedicated SwissProt *Homo sapiens* database (2 July 2020) with an automated false discovery rate. All files from SWATH experiments for cell lines were downloaded to PeakView software and were processed with the previously established library. The resulting data were exported to a.xml file and Marker View software. All data were normalised using the log2 approach and further processed in Perseus software. The mass spectrometry proteomics data were deposited to the ProteomeXchange Consortium via the PRIDE partner repository with the dataset identifier PXD034121 (Perez-Riverol et al., 2019). Cytoscape 3.8.0 (Shannon et al., 2003) and STRING 11.0 (Szklarczyk et al., 2019) were used for the interactome network visualisation, Gene Ontology enrichment analysis, and KEGG pathway analysis for up- and downregulated proteins.

### Pathway enrichment analysis

An open-source Cytoscape 3.9.0 software with ReactomeFIVIz App (Wu et al., 2014a) was employed (accessed 28 February 2022) to perform Pathway Enrichment Analysis. The input data for the Pathway Enrichment Analysis consisted of the gene names of the proteins identified and quantified in the BEAS-2B and A549 cell lines treated with *Dendrobaena veneta* coelomic fluid during the SWATH-MS proteomic experiment, as described above. Then, the corresponding to these proteins Log10 (Fold Change) values for the BEAS-2B and A549 cell lines were overlaid with the significantly enriched pathways. Finally, the resulting pathways were evaluated in the context of the presence of proteins characterised by statistically significant fold change (*p* < 0.05) in cell lines.

## Results

### Inhibitory effect of Venetin-1 on A549 cell viability

**FIGURE 1:**
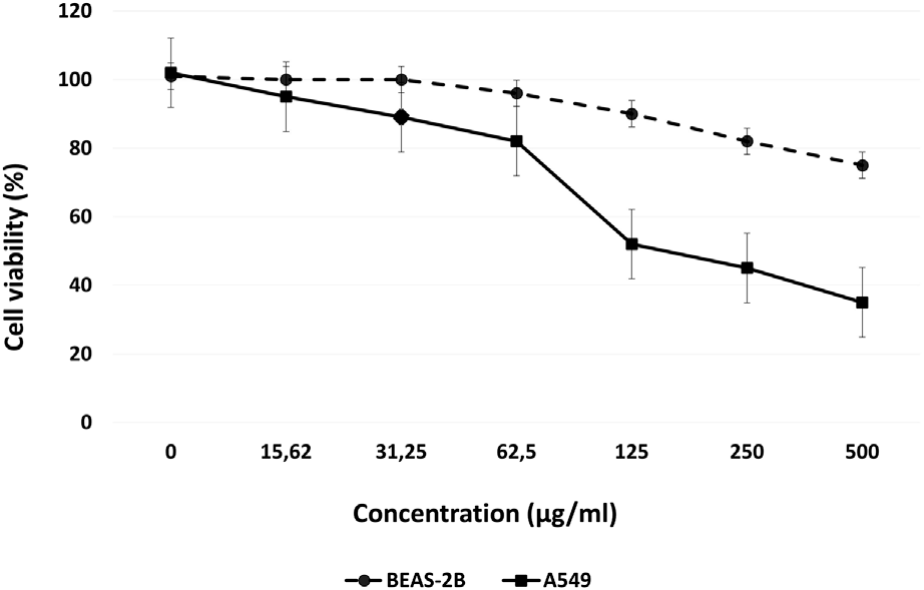
Dose-response curves for Venetin-1 in BEAS-2B and A549 cells, following 72 h incubation.

The effects of various concentrations of Venetin-1 on BEAS-2B and A549 cell proliferation were determined using an MTT assay. The A549 viability rate was significantly decreased to 52%, 41%, and 32% at concentrations of 125 μg/mL, 250 μg/mL and 500 μg/mL, respectively, after 72 h incubation. For the BEAS-2B cell line, only 500 μg/mL concentration affected cell viability (Figure 1).

### Venetin-1 enhanced the cell apoptosis of A549

**FIGURE 2:**
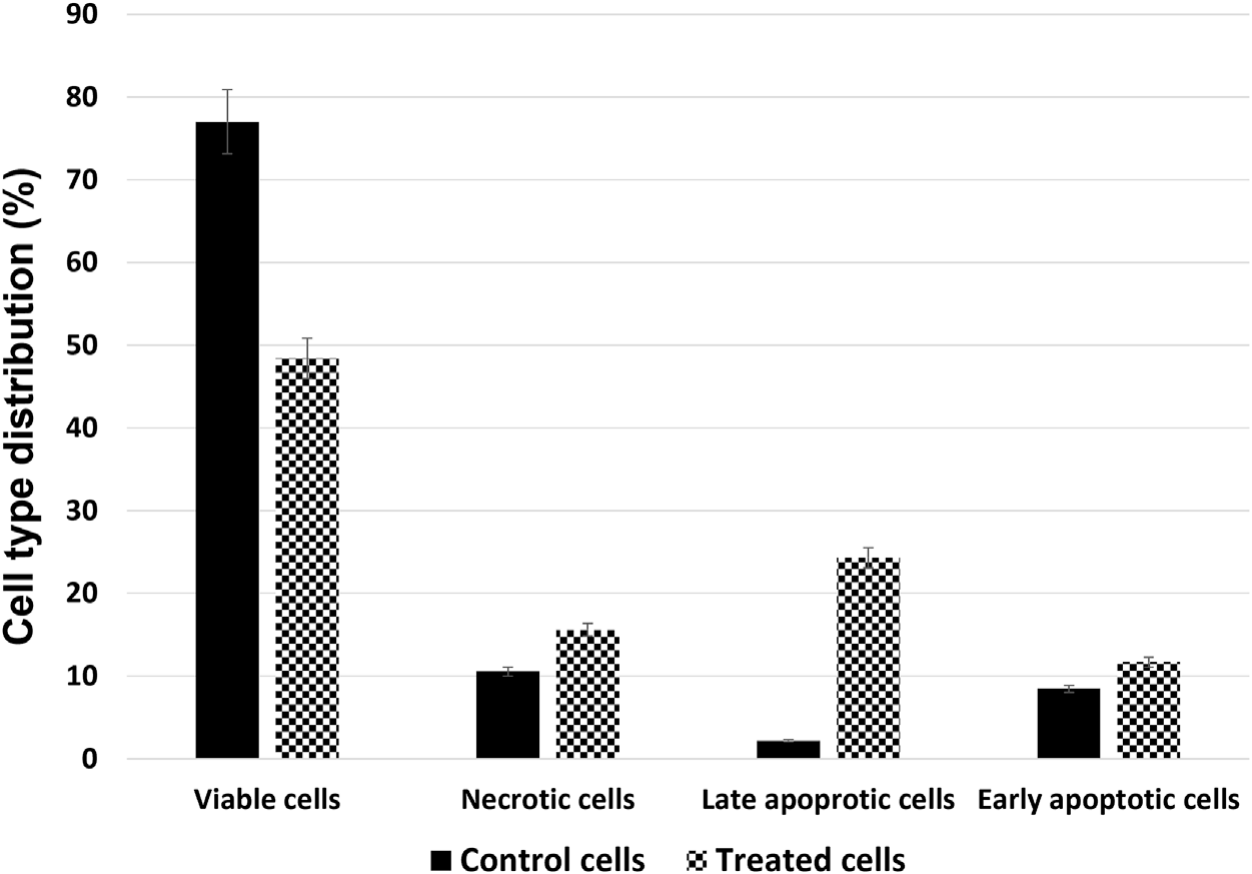
Cell type distribution in the A549 cell line after treatment with 125 μg/mL of Venetin-1 for 72 h.

The amount of apoptotic cell death was quantified with Annexin V-FITC/PI double-labelled flow cytometry. Incubation with 125 μM of Venetin-1 for 72 h increased the amount of apoptosis in this cell line (Figure 2). The total apoptosis rates (late and early apoptosis) have increased significantly compared to untreated cells (36.99 ± 0.57 vs. 10.65 ± 0.27).

### Venetin-1 induced A549 cell autophagy

**FIGURE 3:**
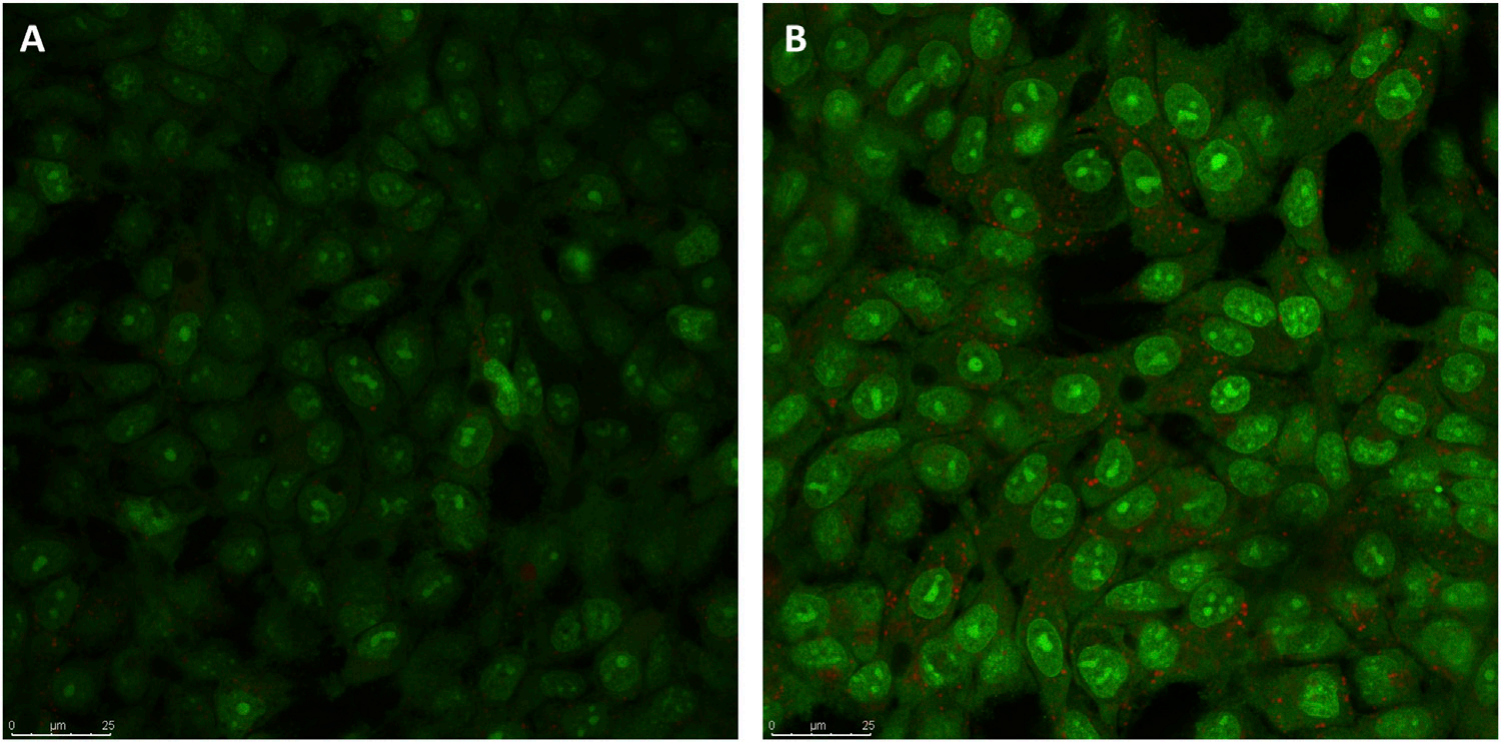
Indication of autophagy by acridine orange staining. **(A)** Representative micrographs of acridine orange staining of A549 control cells **(B)** Representative micrographs of acridine orange staining of A549 cells after treatment with Venetin-1. Acridine Orange staining showed an increase in red fluorescent AVOs within the Venetin-1 treated A549 after 72 h incubation.

In order to verify whether Venetin-1 induced the autophagic pathway, acridine orange staining of live cells was used to visualise the formation of acidic vesicular organelles (AVOs), in both the control and the treated cells. As shown in Figure 3, treating A549 cells with Venetin-1 for 72 h significantly increased the amount of AVOs (red fluorescence). There was no difference in the red fluorescence, which indicates autophagy, between the treated and untreated BEAS-2B cells (Supplementary Figure S2).

### Proteomics

For the proteomic analysis, the BEAS-2B cell lines and the A549 line were treated with Venetin-1. The comparisons were made for Venetin-1 treated versus non-treated BEAS-2B cells (BEAS-2B + v BEAS-2B-) and A549 treated versus A549 control (A549 + v A549-). For the proteomics analyses, we used the FASP method, where, after the cell lysis and release of the proteins, they were digested on a 10 kDa cut-off membrane. The relative quantitative analysis was performed based on the SWATH-MS methodology. A total of 900 proteins for both cell lines were quantified. The obtained data were statistically processed, and based on the obtained q-value (for values <0.05) and fold change (FC), statistically significant changes in protein levels were selected for both cell lines (complete data available in Supplementary Table S1). Assuming the screening criteria as the fold change limit of 1.5 for upregulated proteins and an FC value of 0.67 for downregulated proteins, the most significant changes were selected and presented in Table 1. In the case of changes in the normal cell line, with the statistical assumptions made, no significant changes in protein levels were observed for BEAS-2B cells incubated in the presence of Venetin-1. The proteome response of A549 cells was exemplary. Venetin-1 increased the expression of 31 proteins and decreased level of 22 proteins compared to the self-incubated A549 line.

**TABLE 1:** Up and downregulated protein list for Venetin-1 treated A549 cell line (q-value breakpoint <0.05, FC > 1.5 or <0.67).

	N	Uniprot ID	Gene names	Protein name	q-value	Fold change (A549+/A549-)
**upregulated**	1	P14406	COX7A2	Cytochrome c oxidase subunit 7A2, mitochondrial	0.04	7.68
	2	P11021	HSPA5	Endoplasmic reticulum chaperone BiP	0.00	4.34
	3	Q10713	PMPCA	Mitochondrial-processing peptidase subunit alpha	0.03	3.89
	4	P41252	IARS1	Isolecine-tRNA ligase, cytoplasmic	0.02	2.80
	5	Q9Y4L1	HYOU1	Hypoxia upregulated protein 1	0.03	2.12
	6	P13667	PDIA4	Protein disulfide-isomerase A4	0.03	2.06
	7	Q99714	HSD17B10	3-hydroxyacyl-CoA dehydrogenase type-2	0.03	2.02
	8	O00571	DDX3X	ATP-dependent RNA helicase DDX3X	0.03	2.01
	9	P14625	HSP90B1	Endoplasmin	0.03	1.93
	10	P00403	COX2	Cytochrome c oxidase subunit 2	0.02	1.83
	11	P35232	PHB1 PHB	Prohibitin	0.02	1.76
	12	P61604	HSPE1	10 kDa heat shock protein, mitochondrial	0.02	1.73
	13	P23588	EIF4B	Eukaryotic translation initiation factor 4B	0.02	1.73
	14	P13804	ETFA	Electron transfer flavoprotein subunit alpha, mitochondrial	0.02	1.69
	15	Q96IX5	ATP5MK	ATP synthase membrane subunit DAPIT, mitochondrial	0.02	1.69
	16	P31937	HIBADH	3-hydroxyisobutyrate dehydrogenase, mitochondrial	0.05	1.65
	17	Q56VL3	OCIAD2	OCIA domain-containing protein 2	0.03	1.63
	18	P31930	UQCRC1	Cytochrome b-c1 complex subunit 1, mitochondrial	0.03	1.63
	19	Q15084	PDIA6	Protein disulfide-isomerase A6	0.02	1.57
	20	O00232	PSMD12	26S proteasome non-ATPase regulatory subunit 12	0.03	1.56
	21	P27144	AK4 AK3 AK3L1	Adenylate kinase 4, mitochondrial	0.03	1.55
	22	Q99829	CPNE1 CPN1	Copine-1	0.04	1.55
	23	P0DMV9	HSPA1B HSP72	Heat shock 70 kDa protein 1B	0.04	1.53
	24	Q02878	RPL6 TXREB1	60S ribosomal protein L6	0.03	1.53
	25	O00567	NOP56 NOL5A	Nucleolar protein 56	0.03	1.51
	26	P08183	ABCB1 MDR1	ATP-dependent translocase ABCB1	0.02	1.50
	27	P36542	ATP5F1C	ATP synthase subunit gamma, mitochondrial	0.03	1.49
	28	P47929	LGALS7	Galectin-7	0.03	1.47
	29	P78344	EIF4G2	Eukaryotic translation initiation factor 4 gamma 2	0.04	1.47
	30	P69905	HBA1; HBA2	Hemoglobin subunit alpha	0.03	1.47
	31	Q9H7Z7	PTGES2	Prostaglandin E synthase 2	0.03	1.45
**downregulated**	1	P08758	ANXA5	Annexin A5	0.02	0.69
	2	Q15185	PTGES3	Prostaglandin E synthase 3	0.03	0.68
	3	P00338	LDHA	L-lactate dehydrogenase A chain	<0.01	0.68
	4	P55209	NAP1L1	Nucleosome assembly protein 1-like 1	0.02	0.68
	5	Q13242	SRSF9	Serine/arginine-rich splicing factor 9	0.03	0.67
	6	P29966	MARCKS	Myristoylated alanine-rich C-kinase substrate	0.04	0.65
	7	P18621	RPL17	60S ribosomal protein L17	0.02	0.64
	8	P06744	GPI	Glucose-6-phosphate isomerase	0.02	0.61
	9	Q8NC51	SERBP1	Plasminogen activator inhibitor 1 RNA-binding protein	0.03	0.59
	10	P06454	PTMA	Prothymosin alpha	0.02	0.56
	11	P09972	ALDOC	Fructose-bisphosphate aldolase C	0.03	0.54
	12	Q92597	NDRG1	Protein NDRG1	0.04	0.52
	13	P04075	ALDOA	Fructose-bisphosphate aldolase A	0.00	0.52
	14	Q71UI9	H2AZ2	Histone H2A.V	0.02	0.48
	15	P13674	P4HA1	Prolyl 4-hydroxylase subunit alpha-1	0.03	0.39
	16	P30049	ATP5F1D	ATP synthase subunit delta, mitochondrial	0.04	0.38
	17	P07148	FABP1	Fatty acid-binding protein, liver	0.03	0.32
	18	P02787	TF PRO1400	Serotransferrin	0.04	0.28
	19	P13645	KRT10	Keratin, type I cytoskeletal 10	0.02	0.24
	20	P04264	KRT1	Keratin, type II cytoskeletal 1	0.00	0.12
	21	P35527	KRT9	Keratin, type I cytoskeletal 9	0.00	0.07
	22	P35908	KRT2	Keratin, type II cytoskeletal 2 epidermal	0.04	0.06

**FIGURE 4:**
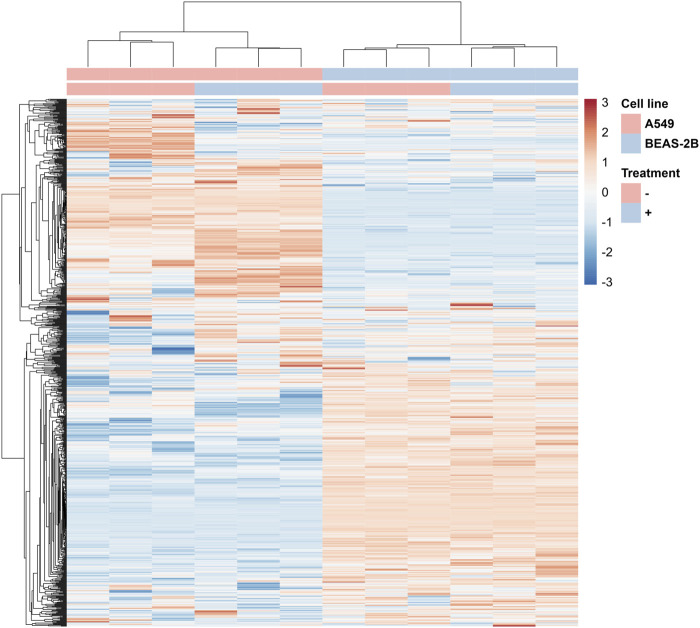
Fragment of the heatmap showing changes in protein levels for A549 cells incubated with Venetin-1 (full heatmap available in the Data Sheet 1).

The changes taking place in A549 cells under the influence of Venetin-1 are also illustrated by the heat map prepared based on the results of the quantitative SWATH analysis (Figure 4). In the clustering heat map, each row represents a protein, and each column corresponds to an analysed sample. Red represents significantly upregulated proteins and blue significantly downregulated ones. For the full-size map and the fully functional analysis for up- and downregulated proteins, (Supplementary Figure S2; Supplementary Table S1).

### Enrichment analysis

For a deeper understanding of the influence of Venetin-1 on cancer cells, GO terms such as biological processes (BP), molecular functions (MF), and subcellular compartments (CC) have been characterised. We used STRING database resources to annotate GO functions for all proteins. A functional analysis was performed for proteins expressed differently as the next step. Complete analysis for proteins showing overexpression and decreased expression is available in the Supplementary Table S2). 89 biological processes for upregulated and 145 for downregulated GO items were matched for treated A549. When overexpressed, BPs are associated mostly with important response processes to protein misfolding, metabolism, oxidative phosphorylation, and stress response (Table 2). In the case of downregulated, these are glycolysis/gluconeogenesis processes related to fructose metabolism, NAD metabolism, ATP, purine ribonucleotides and protein and peptide oligomerisation processes (Table 3).

**TABLE 2:** GO enrichment terms for significantly upregulated proteins in the case of A549 cells in the presence of Venetin-1 (BP–biological processes, MF–molecular function, CC–cellular components).

#category	Term ID	Term description
BP	GO:0006986	Response to unfolded protein
BP	GO:0006457	Protein folding
BP	GO:0046034	ATP metabolic process
BP	GO:0034620	Cellular response to unfolded protein
BP	GO:0061077	Chaperone-mediated protein folding
BP	GO:0034976	Response to endoplasmic reticulum stress
BP	GO:0044237	Cellular metabolic process
BP	GO:0008152	Metabolic process
BP	GO:0051085	Chaperone cofactor-dependent protein refolding
BP	GO:0030968	Endoplasmic reticulum unfolded protein response
BP	GO:0006091	Generation of precursor metabolites and energy
BP	GO:0006119	Oxidative phosphorylation
MF	GO:0009055	Electron transfer activity
MF	GO:0016491	Oxidoreductase activity
MF	GO:0003723	RNA binding
MF	GO:1901363	Heterocyclic compound binding
MF	GO:0097159	Organic cyclic compound binding
MF	GO:0051082	Unfolded protein binding
MF	GO:0033592	RNA strand annealing activity
MF	GO:0015036	Disulfide oxidoreductase activity
MF	GO:0016860	Intramolecular oxidoreductase activity
CC	GO:0005739	Mitochondrion
CC	GO:0034663	Endoplasmic reticulum chaperone complex
CC	GO:0005737	Cytoplasm
CC	GO:0070062	Extracellular exosome
CC	GO:0098798	Mitochondrial protein complex
CC	GO:0005615	Extracellular space
CC	GO:0005743	Mitochondrial inner membrane
CC	GO:0005759	Mitochondrial matrix
CC	GO:0032991	Protein-containing complex
CC	GO:0071682	Endocytic vesicle lumen
CC	GO:0031982	Vesicle
CC	GO:0042470	Melanosome
CC	GO:0031410	Cytoplasmic vesicle
CC	GO:0005576	Extracellular region
CC	GO:0098800	Inner mitochondrial membrane protein complex
CC	GO:0070013	Intracellular organelle lumen
CC	GO:0005788	Endoplasmic reticulum lumen
CC	GO:0043227	Membrane-bounded organelle
CC	GO:0016281	Eukaryotic translation initiation factor 4f complex
CC	GO:0005925	Focal adhesion
CC	GO:0016020	Membrane
CC	GO:0031967	Organelle envelope
CC	GO:0070469	Respirasome
CC	GO:0005753	Mitochondrial proton-transporting ATP synthase complex
CC	GO:1990904	Ribonucleoprotein complex
CC	GO:1904813	ficolin-1-rich granule lumen

**TABLE 3:** GO enrichment terms for significantly downregulated proteins in the case of A549 cells in the presence of Venetin-1 (BP–biological processes, MF–molecular function, CC–cellular components).

#category	Term ID	Term description
BP	GO:0006096	Glycolytic process
BP	GO:0019674	NAD metabolic process
BP	GO:0046034	ATP metabolic process
BP	GO:0061621	Canonical glycolysis
BP	GO:0070268	Cornification
BP	GO:0018149	Peptide cross-linking
BP	GO:0097435	Supramolecular fibre organisation
BP	GO:0016043	Cellular component organisation
BP	GO:0009150	Purine ribonucleotide metabolic process
BP	GO:0006094	Gluconeogenesis
BP	GO:0030388	Fructose 1,6-bisphosphate metabolic process
BP	GO:0006091	Generation of precursor metabolites and energy
BP	GO:0045055	Regulated exocytosis
BP	GO:0051262	Protein tetramerisation
BP	GO:0006000	Fructose metabolic process
BP	GO:0051290	Protein heterotetramerization
BP	GO:0043933	Protein-containing complex subunit organisation
MF	GO:0030280	Structural constituent of skin epidermis
MF	GO:0004332	Fructose-bisphosphate aldolase activity
CC	GO:0070062	Extracellular exosome
CC	GO:0031982	Vesicle
CC	GO:1904813	ficolin-1-rich granule lumen
CC	GO:0001533	Cornified envelope
CC	GO:0043227	Membrane-bounded organelle
CC	GO:0005829	Cytosol
CC	GO:0005882	Intermediate filament
CC	GO:0005634	Nucleus
CC	GO:0072562	Blood microparticle
CC	GO:0043231	Intracellular membrane-bounded organelle
CC	GO:0034774	Secretory granule lumen
CC	GO:0099512	Supramolecular fiber

**FIGURE 5:**
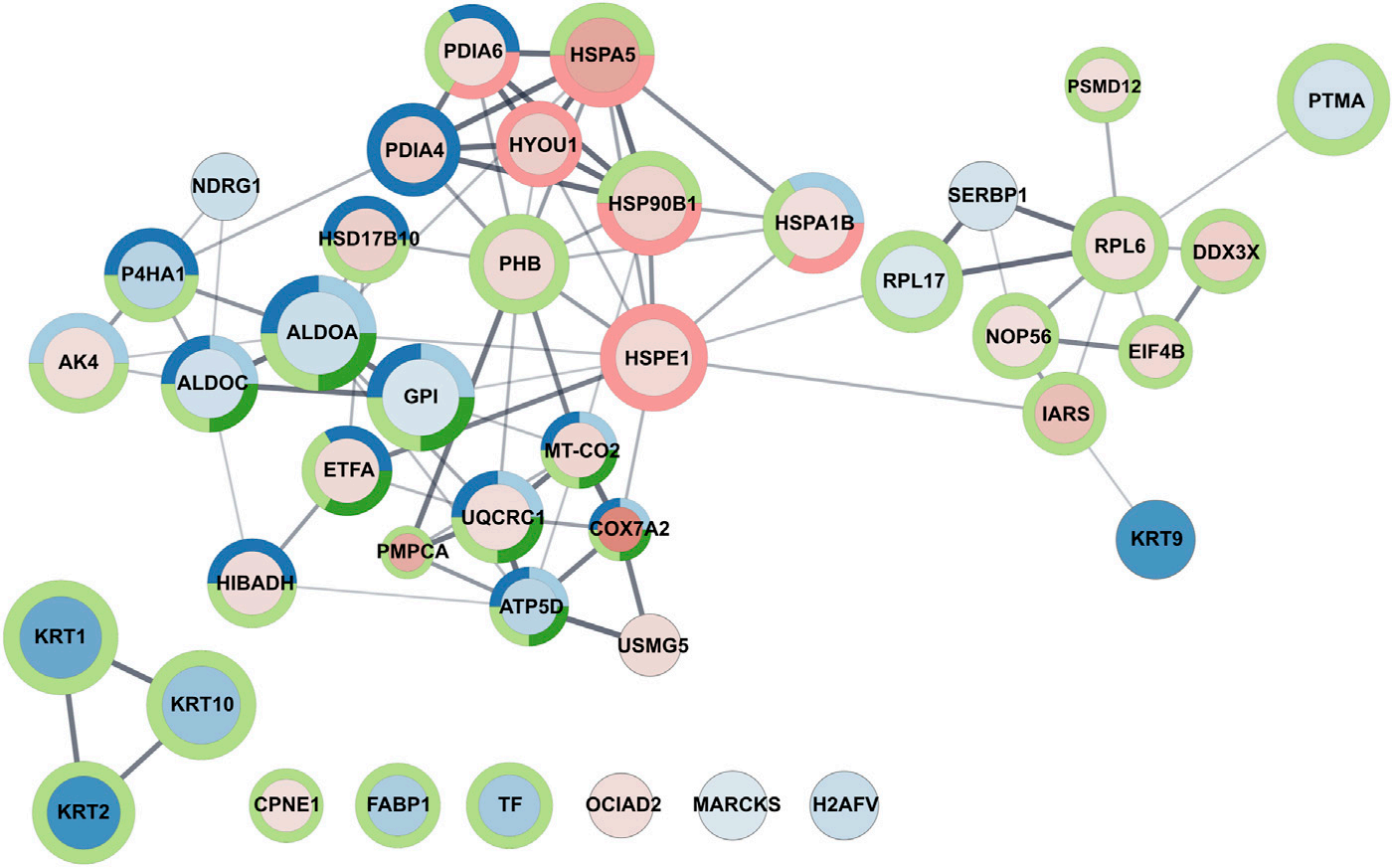
Graphic representation of GO enrichment in the biological process category for A549 cell line treated with Venetin-1. The size of the circle and colour intensity corresponds to the fold change. Fill-in colors refer to red - upregulated proteins and blue - downregulated proteins, respectively. The outer circles relate to functional analysis, and its colours correspond to Uniprot keywords enrichment light blue: ATP metabolic process, dark blue: oxidation-reduction process, light green: cellular metabolic process, dark green: generation of precursor metabolites and energy, red: response to unfolded protein.

As an example of GO enrichment in biological processes for significantly downregulated proteins, Cytoscape graphic representation (Figure 5) was prepared to consider the multiplicity of changes and the processes in which proteins are involved.

KEGG pathways enrichment analysis for A549 cells (Table 4, complete data in Supplementary Table S2) revealed 23 enriched pathways for upregulated proteins and 9 for downregulated ones. In the table, they were sorted in ascending order based on FDR and *p*-value. The first five cases of upregulated for Venetin-1 treated A549 cells versus controls (untreated line A549) concern pathways associated with Parkinson, Prion, Alzheimer, Huntington, and Amyotrophic lateral sclerosis. The remaining 18 pathways are more characteristic of cancer lines. Reduced activity was observed for glycolysis/gluconeogenesis, carbon metabolism, amino acid biosynthesis, HIF-1 signalling pathways, pentose phosphate pathways, metabolic, ribosome, fructose and mannose metabolism and estrogen signalling pathway.

**TABLE 4:** KEGG Pathways for A549 cells treated with Venetin-1 for up- and downregulated proteins.

Upregulated					Downregulated				
Term name	# Genes	Description	*p*-value	FDR value	Term name	# Genes	Description	*p*-value	FDR value
hsa05012	15	Parkinson disease	6.33E-15	2.88E-12	hsa00010	8	Glycolysis/Gluconeogenesis	3.53E-11	1.6E-8
hsa05020	14	Prion disease	4.94E-13	1.12E-10	hsa01200	8	Carbon metabolism	2.75E-9	6.25E-7
hsa05010	13	Alzheimer disease	2.91E-10	4.4E-8	hsa01230	7	Biosynthesis of amino acids	3.21E-9	6.25E-7
hsa05016	12	Huntington disease	5.36E-10	6.09E-8	hsa04066	6	HIF-1 signalling pathway	8.65E-7	9.82E-5
hsa05014	11	Amyotrophic lateral sclerosis	3.78E-8	3.43E-6	hsa00030	4	Pentose phosphate pathway	2.46E-6	2.2E-4
hsa00190	7	Oxidative phosphorylation	4.24E-7	3.21E-5	hsa01100	16	Metabolic pathways	2.59E-6	2.2E-4
hsa05017	7	Spinocerebellar ataxia	5.4E-7	3.5E-5	hsa03010	6	Ribosome	2.69E-6	2.2E-4
hsa01100	18	Metabolic pathways	1.28E-6	7.24E-5	hsa00051	3	Fructose and mannose metabolism	1.4E-4	0.0081
hsa04141	7	Protein processing in endoplasmic reticulum	1.96E-6	9.88E-5	hsa04915	4	Estrogen signalling pathway	6.7E-4	0.034
hsa04714	7	Thermogenesis	1.56E-5	7.1E-4					
hsa01200	5	Carbon metabolism	6.06E-5	0.0025					
hsa01230	4	Biosynthesis of amino acids	1.4E-4	0.0053					
hsa00020	3	Citrate cycle (TCA cycle)	1.8E-4	0.0061					
hsa00630	3	Glyoxylate and dicarboxylate metabolism	1.9E-4	0.0061					
hsa04217	5	Necroptosis	1.8E-4	0.0061					
hsa04932	5	Non-alcoholic fatty liver disease	1.8E-4	0.0061					
hsa04979	3	Cholesterol metabolism	7.0E-4	0.0188					
hsa05134	3	Legionellosis	0.001	0.0259					
hsa03010	4	Ribosome	0.0011	0.0272					
hsa00730	2	Thiamine metabolism	0.0013	0.0303					
hsa04612	3	Antigen processing and presentation	0.0015	0.0322					
hsa01210	2	2-Oxocarboxylic acid metabolism	0.0021	0.0432					
hsa04918	3	Thyroid hormone synthesis	0.0023	0.0458					

**FIGURE 6:**
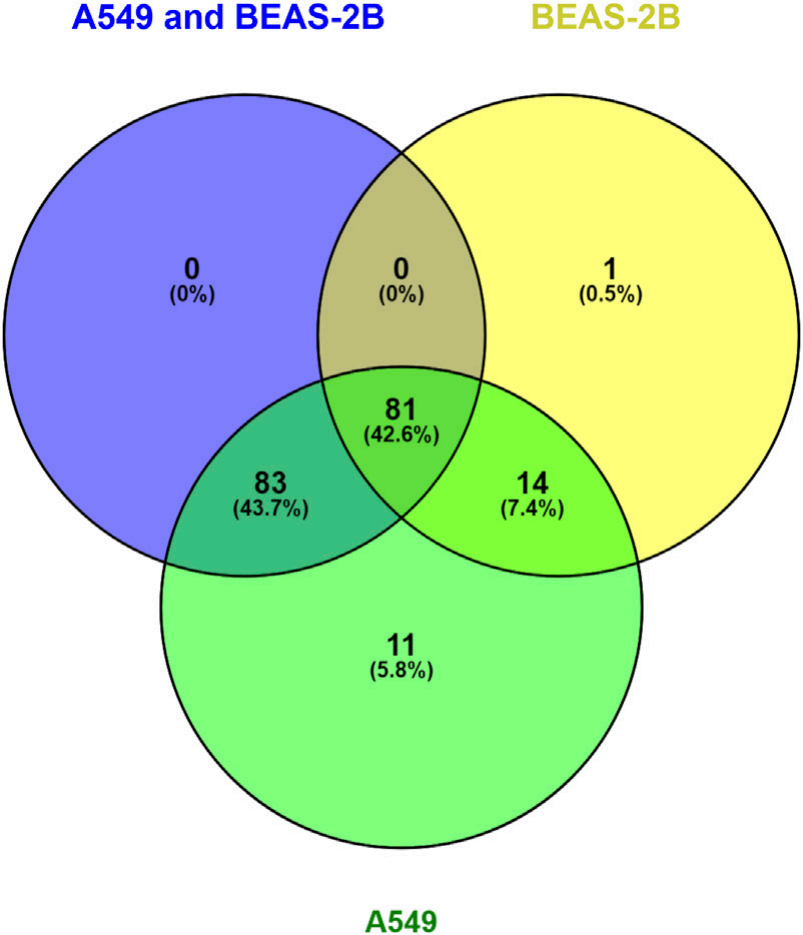
Venn Diagram compares the molecular pathways statistically significantly enriched in reactome analysis for A549 and BEAS-2B treated with Venetin-1.

We performed a Pathway Enrichment analysis using Cytoscape 3.9.0 software with ReactomeFIVIz App (Wu et al., 2014a) to integrate, evaluate and visualise functional relationships between the detected and quantified proteins in BEAS-2B and A549 cell lines in response to the *Denadrobaena veneta* coelomic fluid. Such projection of the proteomic data onto Reactome pathways context (Gillespie et al., 2022) enabled us to obtain a comprehensive view of the processes affected by the *Denadrobaena veneta* coelomic fluid treatment. The analysis resulted in the statistically significant (*p* < 0.001) enrichment of 189 pathways at the FDR level of <0.001. Then, the information about statistically significant fold changes of proteins detected in A549, BEAS-2B, and both A549 and BEAS-2B cell lines in response to *Denadrobaena veneta* coelomic fluid was overlaid with the selected statistically significantly enriched pathways. As a result, we found that in all 189 statistically significantly enriched pathways. There were proteins (genes) characterised by statistically significant fold change in the A549 cell line (Supplementary Table S3. Pathway Enrichment Analysis Results). There were 164 statistically significantly enriched pathways, which included proteins of statistically significant fold change in both A549 and BEAS-2B cell lines (Supplementary Table S3. Pathway Enrichment Analysis Results). We found that there were 96 statistically significantly enriched pathways, in which there were assigned proteins with the statistically significant fold change in BEAS-2B cell line (Supplementary Table S3. Pathway Enrichment Analysis Results). Then, we compared the pathways in which there were included proteins of statistically significant fold change detected in A549, BEAS-2B and both A549 and BEAS-2B cell lines (Figure 6).

We observed that there was only one statistically significantly enriched pathway, namely, Nucleobase Biosynthesis (*p* = 0,00011263; FDR = 0,00056316), in which there were only proteins of statistically significant fold change detected in the BEAS-2B cell line (Figure 5). In turn, there were 11 molecular pathways statistically significantly enriched in our dataset, where significant protein fold change was observed only in the A549 cell line upon treatment with *Dendrobaena veneta* celomic fluid (Figure 4).

## Discussion

Malignant cells are known to reprogram their metabolism and protein expression to increase proliferation and invasiveness. One of the most extensively described changes for most cancers is the transition to glucose metabolism by lactic acid fermentation (anaerobic glycolysis), even in the presence of oxygen, the so-called Warburg effect. It is not different in the case of lung cancer cells. However, the molecular targets through which Venetin-1 acts on human cells have not been determined yet. In this study, we make the first attempt to use proteomics and bioinformatic tools to determine the anti-cancer nature of the preparation. In the present proteomic studies, we compared the effect of Venetin-1 fraction on proteins expression levels in 2 cell lines, BEAS-2B normal epithelial cells (BEAS v BEAS + Venetin-1) and A549 lung cancer cell line (A549 v A549 + Venetin-1). The first crucial observation is that the preparation did not significantly affect BEAS-2B cells. The analysis did not show any statistically significant changes in protein levels, which means that the proteome response of normal cells to the components contained in Venetin-1, at this point, can be classified as insignificant.

In contrast to normal cells, the A549 tumour line proteome responded to the presence of 31 upregulated proteins (q < 0.05, FC > 1.5) and 18 downregulated proteins (q < 0.05, FC < 0.67) (Table 1). Proteins with increased expression in neoplastic cells are mainly associated with the mitochondrion, membrane transport and the endoplasmic reticulum. It is confirmed in the analysis of the reactome (Table 5), where the cell reaction manifests itself in the form of 11 enriched pathways such as, i.e., cross-presentation of soluble exogenous antigens (endosomes), regulation of ornithine decarboxylase (ODC), metabolism of polyamines, ER to Golgi Anterograde Transport, COPI-mediated anterograde transport, Interconversion of nucleotide di- and triphosphates, Beta oxidation of octanoyl-CoA to hexanoyl-CoA, Golgi-to-ER retrograde transport, mitochondrial fatty acid beta-oxidation of unsaturated fatty acids, beta-oxidation of decanoyl-CoA to octanoyl-CoA-CoA, transport to the Golgi and subsequent modification. In the case of altered proteins, Venetin-1 interferes with proteins that stabilise the structures, i.e., keratin, glycolysis/gluconeogenesis and metabolic processes.

**TABLE 5:** Molecular pathways statistically significantly enriched in reactome analysis for A549 cells.

	Reactome Pathway	Ratio Of Protein In Pathway	Number Of Protein In Pathway	Protein From Gene SET	Number of proteins with statistically significant FC in A549 in the pathway	Number of proteins with statistically significant FC in A549 and BEAS-2B S in the pathway	Number of proteins with statistically significant FC in BEAS-2B in the pathway	*p*-value	FDR	HitGenes (all the significant FC marked as bold)
1	Cross-presentation of soluble exogenous antigens (endosomes)	0,0046	50	28	3	0	0	1,11E-16	2,44E-15	PSMD5, PSMD2, PSMD3, PSMD1, PSME1, PSME2, PSMA5, PSMA6, PSMA3, PSMA1, PSMA2, PSMA7, PSMB7, PSMB4, PSMB5, PSMB2, PSMB3, PSMB1, PSMC5, PSMC6, PSMC3, PSMC4, PSMC1, PSMC2, PSMD12, PSMD11, PSMD14, PSMD13
2	Regulation of ornithine decarboxylase (ODC)	0,0047	51	29	4	0	0	1,11E-16	2,44E-15	PSMD5, PSMD2, PSMD3, PSMD1, PSME1, PSME2, PSMA5, PSMA6, PSMA3, PSMA1, PSMA2, PSMA7, PSMB7, PSMB4, PSMB5, PSMB2, PSMB3, PSMB1, PSMC5, PSMC6, PSMC3, PSMC4, PSMC1, PSMC2, PSMD12, PSMD11, PSMD14, PSMD13, NQO1
3	Metabolism of polyamines	0,0054	59	31	4	0	0	1,11E-16	2,44E-15	PSMD5, PSMD2, PSMD3, PSMD1, PSME1, PSME2, PSMA5, PSMA6, PSMA3, PSMA1, PSMA2, PSMA7, PSMB7, PSMB4, PSMB5, PSMB2, PSMB3, PSMB1, PSMC5, PSMC6, PSMC3, PSMC4, PSMC1, PSMC2, SRM, PSMD12, PSMD11, PSMD14, PSMD13, AGMAT, NQO1
4	ER to Golgi Anterograde Transport	0,0124	136	26	6	0	0	6,95E-06	4,17E-05	ARF4, ARF1, DYNLL2, ARCN1, CAPZB, SEC22B, SEC23A, SEC31A, ACTR1A, SPTAN1, SEC13, DYNC1H1, TMED10, COPZ1, USO1, LMAN1, LMAN2, SCFD1, SPTBN1, TMED2, TMED7, COPG1, NSF, COPB2, COPA, COPB1
5	COPI-mediated anterograde transport	0,0076	83	19	5	0	0	1,08E-05	6,47E-05	ARF4, ARF1, DYNLL2, ARCN1, CAPZB, ACTR1A, SPTAN1, DYNC1H1, TMED10, COPZ1, USO1, SPTBN1, TMED2, TMED7, COPG1, NSF, COPB2, COPA, COPB1
6	Interconversion of nucleotide di- and triphosphates	0,0027	30	11	6	0	0	1,28E-05	7,70E-05	GSR, AK1, AK2, AK4, GLRX, TXNRD1, TXN, GUK1, NME2, NME1, CMPK1
7	Beta oxidation of octanoyl-CoA to hexanoyl-CoA	0,0005	5	5	2	0	0	3,30E-05	1,98E-04	ECHS1, ACADM, HADHB, HADHA, HADH
8	Golgi-to-ER retrograde transport	0,0107	117	22	4	0	0	4,39E-05	2,63E-04	ARF4, ARF1, DYNLL2, PAFAH1B3, PAFAH1B2, ARCN1, CAPZB, SEC22B, SURF4, ACTR1A, DYNC1H1, TMED10, COPZ1, KLC1, RAB6A, TMED2, TMED7, COPG1, NSF, COPB2, COPA, COPB1
9	Mitochondrial fatty acid beta-oxidation of unsaturated fatty acids	0,0005	6	5	3	0	0	7,74E-05	4,65E-04	ECI1, DECR1, ACADM, HADHB, HADHA
10	Beta oxidation of decanoyl-CoA to octanoyl-CoA-CoA	0,0005	6	5	2	0	0	7,74E-05	4,65E-04	ECHS1, ACADM, HADHB, HADHA, HADH
11	Transport to the Golgi and subsequent modification	0,0153	167	26	6	0	0	1,94E-04	9,68E-04	ARF4, ARF1, DYNLL2, ARCN1, CAPZB, SEC22B, SEC23A, SEC31A, ACTR1A, SPTAN1, SEC13, DYNC1H1, TMED10, COPZ1, USO1, LMAN1, LMAN2, SCFD1, SPTBN1, TMED2, TMED7, COPG1, NSF, COPB2, COPA, COPB1

### Venetin-1 hits the activity of the mitochondria and the endoplasmic reticulum

Analysing the results obtained for upregulated proteins, it can be noticed that the changes caused by the presence of the earthworm preparation cause a response in i) the mitochondrion and its inner membrane, ii) endoplasmic reticulum (ER), iii) ER unfolded protein response (UPR) and iv) RNA binding. Among the proteins associated with mitochondrial activity, we can mention Cox7A2, PMPCA, Cox2, ATP5MK, UQCRC1, ATPF1C and PTGES2. In the case of cytochrome C oxidase subunit 7A2, mitochondrial (Cox7A2), the elevation is almost 8-fold (FC 7.7) in Venetin-1 treated A549 cells as compared to A549 cells. The change is fourfold for the protein mitochondrial-processing peptidase subunit alpha (PMPCA) (FC 3.9). Mitochondrial dysfunction in cancer cells is a well-known phenomenon (Wang et al., 2008), but here we have an additional increase in protein levels due to the presence of Venetin-1. Cox7A2 is a protein part of the complex IV of the electron transport chain (ETC.) (Galati et al., 2009). COX7A2 is required to maintain the expected level of COX activity.

In cancer, the prognosis associated with elevated or lowered protein levels may be associated with both positive and negative prognoses for patients and must be verified each time. In the case of Cox7A2, which has not been well characterised in the context of cancer, the literature data available for glioma patients indicate that overexpression levels of this protein are associated with good prognosis of patients (Deng et al., 2018). The lung cancer data, specifically non-small cell lung cancer, the Cox7A1 isoform was investigated, and its elevated level suppresses cell proliferation and colony formation and promotes apoptosis (Zhao et al., 2019). The second protein from this group is mitochondrial-processing peptidase subunit alpha (PMPCA), which, together with PMPCB, creates a Mitochondrial Processing Protease MPP (Teixeira and Glaser, 2012; Poveda-Huertes et al., 2017). In this case, we observed almost a fourfold increase (FC 3.9) of expression in Venetin-1 treated cells. The task of the entire MPP complex in the mitochondrion is proteolytic processing of precursor proteins imported to the mitochondrion. The dysfunction of MPP means the accumulation of not functional protein precursors in the mitochondrial matrix. There is no data in the literature on changes in levels in the complex subunits. However, many tests indicate that mutations in the PMPCA sequence cause incorrect protein processing in mitochondrion (Jobling et al., 2015) and severe mitochondrial diseases (Joshi et al., 2016). In our case, we can only speculate that overexpression of one of the subunits of the MPP complex disturbs its work and protein proceeds in the mitochondrion for active forms.

Isolucine-tRNA ligase (IARS1, ILERS OR IRS), for which FC in the conducted studies is three times increased in A549 cells after treatment with the preparation, is one of the 20 aminoacyl-tRNA synthetases. It is also a contraption of a larger MARS complex (multi-aminoacyl-synthetase).

The introduction of Venetin-1 into the environment of the A549 cells results in significant changes at the endoplasmic reticulum level, where proteins like chaperons mediate a significant part of the glycosylation and secretory protein folding. In our results, it manifests itself in increased heat shock proteins (HSPs) such as HSPA5, HSP90B1, HSPE1, HSP72 and related proteins HYOU1, PDIA4 and PDIA6. The fold change values for this group are, on average, doubled, and the endoplasmic reticulum chaperon BiP (HSPA5) is distinguished among them with the FC value 4.3. This chaperone, also known as HSP70 or GRP78, is a stress protein produced by cells. In hypoxia, it is overexpressed (for example, by ER stress), which is typical of cancer cells. Its effect on tumour development is tissue-dependent again. For example, overexpression in ovarian cancer and gastric and renal carcinoma has been associated with increased metastasis and poor diagnosis for patients (Zhang et al., 2006; Kuroda et al., 2011; Huang et al., 2012; Wu et al., 2014b). In the results presented by Sun and co-owners, HSPA5 is overexpressed in cell line A549, promoting hypoxia-induced epithelial-mesenchymal transition (EMT) (Sun et al., 2019). The elevated level of this ER chaperone was also observed in the blood of patients with lung cancer (Ercan et al., 2021). In lung cancer, reports indicate decreased HSPA5 expression with Aftanib by measuring the level of HSPA5 mRNA before and after treatment (Pancewicz-Wojtkiewicz and Bernatowicz, 2017). Other work on the A549 cell line showed that HSPA5 overexpression combined with cisplatin treatment causes early and increased apoptosis by NF-κB activation in ER stress-induced apoptosis, which is further mediated by c-Jun hyperactivation (Ahmad et al., 2014). In contrast, research on the mouse lung cancer model appears, where it has been shown that overexpression of this protein is associated with poor forecasts in therapy, and HSPA5 inhibition by HA15 promoted the apoptosis of tumour cells (Wu et al., 2020).

### Venetin-1 and autophagy

One of the essential degradation pathways of molecules or entire cellular organelles is the process of autophagy. Many natural compounds such as curcumin, resveratrol, plumbagin in traditional medicine were shown to act through this process (Cui and Yu, 2018). In the case of Venetin-1, this effect was observed in *Candida albicans* cells (manuscript submitted to Scientific Reports). Staining with acridine orange showed the presence of characteristic autophagic bodies in A549 cells treated with Venetin-1 (Figure 3). This effect was not seen with the control line BEAS-2B (Supplementary Figure S2). The analysis of proteomic data and the Reactome for the process of autophagy tentatively indicates that among the three autophagy pathways described in the literature (macroautophagy, microautophagy and chaperone-mediated autophagy) (Kaushik and Cuervo, 2012) it may be the process of chaperone-mediated autophagy. This process is studied and described for cancer cells; however, it requires more profound research because changes in the levels of proteins involved in this process are not unambiguous and should be considered individually for a given cancer (Rios et al., 2020). Our preliminary results in this field are the first premises that open another research path leading to a more thorough examination and description of the autophagy process in A549 cells under the influence of Venetin-1.

### Venetin-1 alters the level of structural proteins and affects glycolysis/gluconeogenesis

Cancer line analysis indicated significant downregulation of five cytoskeleton proteins, keratins (KRT2, KRT9, KRT1, KRT10). They belong to both types I (low-molecular-weight acidic and high molecular weight primary type, KRT9, 10) and II (neutral type, KRT2, 1) (Elbalasy et al., 2021; Ho et al., 2022). KRT 1/2 and KRT 10 form heterocomplexes; in our case, all three are down-expressed (Ho et al., 2022). Lowering their expression in A459 cells may negatively affect many processes like cell cycle regulation, the activation of growth factors, apoptosis, cellular response to stress, activation of growth factors, and the structure of the cytoskeleton itself. In the case of biological processes, the reduction of keratin levels is reflected in processes related to cornification, formation of protein complexes, peptides, cross-linking and organisation of cellular components (Table 3).

Glycolysis/gluconeogenesis is a process considered a hallmark of cancer cells’ rapid proliferation (Nowak et al., 2018). In the case of A549 cells treated with Venetin-1, glucose-6-phosphate isomerase (GPI), fructose-bisphosphate aldolase A and B (ALDOA, ALDOB) levels were significantly downregulated. The first protein is the crucial enzyme for the second step of glycolysis but is also involved in the pentose phosphate pathway and glucose synthesis (Tsutsumi et al., 2004), and it can also behave as a potent cytokine and autocrine motility factor. Aldolases regulate the reversible cleavage of fructose 1,6-bisphosphate into glyceraldehyde 3-phosphate. Aldolases regulate the fourth stage of glycolysis associated with the transition from six-carbon to three-carbon molecules (reversible cleavage of fructose 1,6-bisphosphate into glyceraldehyde 3-phosphate). In the case of lung adenocarcinoma (Lu et al., 2021), Lu and co-workers observed that upregulation of ALDOA correlates with tumour progression and poor survival. It is also worth paying attention to the L-lactate dehydrogenase A chain (LDHA), which is a crucial enzyme in aerobic glycolysis (Feng et al., 2018), and in our research, its FC value of 0.68 is slightly above the assumed fold change value (<0.67). So here we influence the presence of the preparation on three enzymes crucial for respiratory processes, which act at three different stages of the transformation of glucose into lactate. Literature data report that in the case of various types of cancers, including lung cancer cells, overexpression of each increases metastatic properties and is associated with poor prognosis for patients (Peng et al., 2012; Lessa et al., 2013; Chen et al., 2014; Zhang et al., 2017; Nowak et al., 2018; Chang et al., 2019; Han et al., 2021; Tu et al., 2021; Wang et al., 2022). Performed KEGG pathway enrichment analysis for downregulated proteins showed enrichment of function for glycolysis/gluconeogenesis, HIF-1 carbon metabolic pathway, pentose phosphate pathway, metabolic pathway, and fructose and mannose metabolism (Table 4). According to the literature data, the HIF-1 signalling pathway and glycolysis/gluconeogenesis play an essential role in tumour invasion and metastasis (Rankin and Giaccia, 2016). Consistent with our results, Venetin-1 downregulates the level of critical enzymes, which may slow down or inhibit the invasion and metastasis of A549 cells.

### Venetin-1 affects proteins related to radio and chemosensitivity

Among the proteins identified in the study with reduced expression, a few are mentioned in the literature concerning sensitising cancer cells to chemotherapy, radiotherapy, or targeted therapies. In our case, this is: histone H2A.V (H2AZ2), prothymosin alpha (PTMA), and N-myc downstream-regulated protein (NDRG1). In the case of H2AZ1, increased expression in cervical cancer, metastatic melanoma or breast cancer is associated with the promotion of the pro-oncogenic transcriptome (activation of genes related to the cell cycle, DNA replication) (Vardabasso et al., 2015; Salmerón-Bárcenas et al., 2021). In the case of this protein, Vardabasso and colleagues postulate that silencing this histone variant makes melanoma cells more sensitive to chemo- and targeted therapy (Vardabasso et al., 2015). In the case of prothymosin alpha (PTMA) protein, in which overexpression indicates a poor prognosis for most cancers, its inhibition described in non-small cell lung cancer allows cells to be sensitised to radiation therapy (Jiang et al., 2021). NDRG1 is a tumour-associated gene that belongs to the superfamily alpha/beta hydrolases (Qu et al., 2002). Depending on the type of tumour, its increased or decreased expression is associated with a poor prognosis for the patient. In the case of lung cancer, including lung adenocarcinoma, overexpression is a weaker prognosis for the patient (Azuma et al., 2012; Dai et al., 2018). Overexpression of NDRG1 significantly downregulated chemosensitivity to cisplatin in lung cancer A549 cells; at the same time, the author informs that overexpression of NDRG1 in lung cancer A549 cells significantly downregulated cisplatin-induced cytotoxicity (Du et al., 2018).

Myristoylated Alanine-Rich C Kinase Substrate (MARCKS), the primary substrate of protein kinase C (PKC) (Blackshear, 1993) is also among the downregulated proteins in A549 cells treated with Venetin-1. MARCKS is involved, *inter alia*, in the cell in control of cytoskeleton, chemotaxis and motility, and mediation of inflammatory response. Cancer contributes to tumorigenesis and metastasis, and its phosphorylated forms play a different role. Depending on the type of cancer, they can enhance or reduce the proliferation and migration of neoplastic cells (Fong et al., 2017; Sheats et al., 2019; Liang et al., 2020). MARCKS levels are elevated in lung cancer and are associated with increased proliferation, migration and invasiveness. It also causes resistance to treatments such as radiation and drug therapy. The overall elevated MARCKS level is again associated with poor prognosis for patients.

The last of the proteins mentioned is plasminogen activator inhibitor 1 RNA-binding protein (SERBP1). It is a serpin mRNA binding protein 1 associated with high tumour development and metastasis potential. The studies so far indicate that cells showing resistance to cis-platinum show the overexpression of SERBP1.

Another protein extensively researched as a potential biomarker in recent years is prolyl 4-hydroxylase, alpha polypeptide I (P4HA1). It is a crucial enzyme in collagen synthesis, an essential element of the tumour microenvironment. The literature has confirmed that P4HA1 expression is increased in many types of cancer (Zhao and Liu, 2021). In lung tumours, data with the above expression are available for lung adenocarcinoma and lung squamous cell carcinoma (Ning et al., 2021). In the case of cancer cells, the increased level of this protein is associated with the promotion of cell proliferation, invasiveness, and migration. In our research, P4HA1 belongs to the group of downregulated proteins, which, according to the literature data, may indicate the possibility of inhibiting cell proliferation, migration and invasion (Ning et al., 2021). This protein may also have sensitising potential, as seen in triple-negative breast cancer (Zhou et al., 2017). There is no literature data on the influence of P4AH1 on the sensitivity of cells during the therapies for lung cancer.

## Conclusion

Considering often opposite literature reports on cancer markers, we are aware that the presented results are limited to one cell line, A549. In order to properly determine the potential of Venetin-1, it must be checked on other cancer lines and in *in-vivo* studies on animal models as the next step.

Thanks to the use of the SWATH-MS methodology allowing for the quantitative analysis of the proteome response of the BEAS-2B and A549 cells to the presence of the Venetin-1 preparation derived from the earthworm, we found that this preparation did not have a significant effect on the proteome of a normal cell line. In contrast, cells from the neoplastic line (A549-v A549 +) of lung cancer strongly respond to its presence by overexpressing proteins related to the activity of the mitochondrion and the endoplasmic reticulum. The levels of proteins associated with glycolysis/gluconeogenesis and the levels of the four keratins are decreased. Interestingly, we observed a reduced expression of several proteins associated with radio-sensitisation and chemotherapy of cancer cells.

Our results must be validated with other methods like immunoassay ELISA or targeted mass spectrometry with patients’ samples. The second thing is that to obtain a complete picture of protein changes and select them as biomarkers, the correlation of research results is needed at the genetic and proteomic change levels.

## Data Availability

The mass spectrometry proteomics data were deposited to the ProteomeXchange Consortium via the PRIDE partner repository with the dataset identifier PXD034121.
