# Supplementary figures and images for: Proteomic response of A549 lung cancer cell line to protein-polysaccharide complex Venetin-1 isolated from earthworm coelomic fluid
Source: Front Mol Biosci. 2023 Jun 8;10:1128320. doi: 10.3389/fmolb.2023.1128320 (PMC10292018; doi:10.3389/fmolb.2023.1128320)

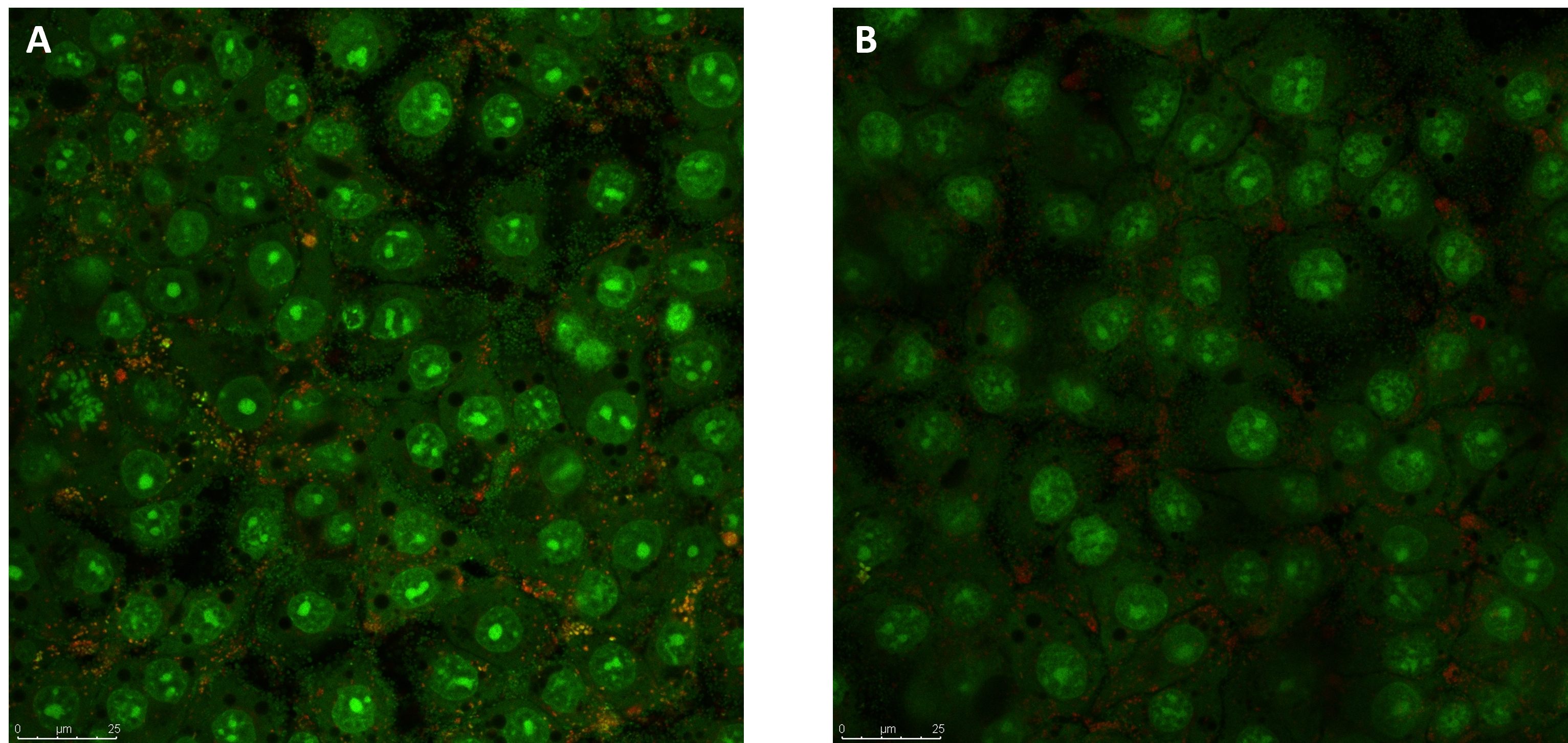

Supplement: Supplementary file 3 [file Image2.TIF]

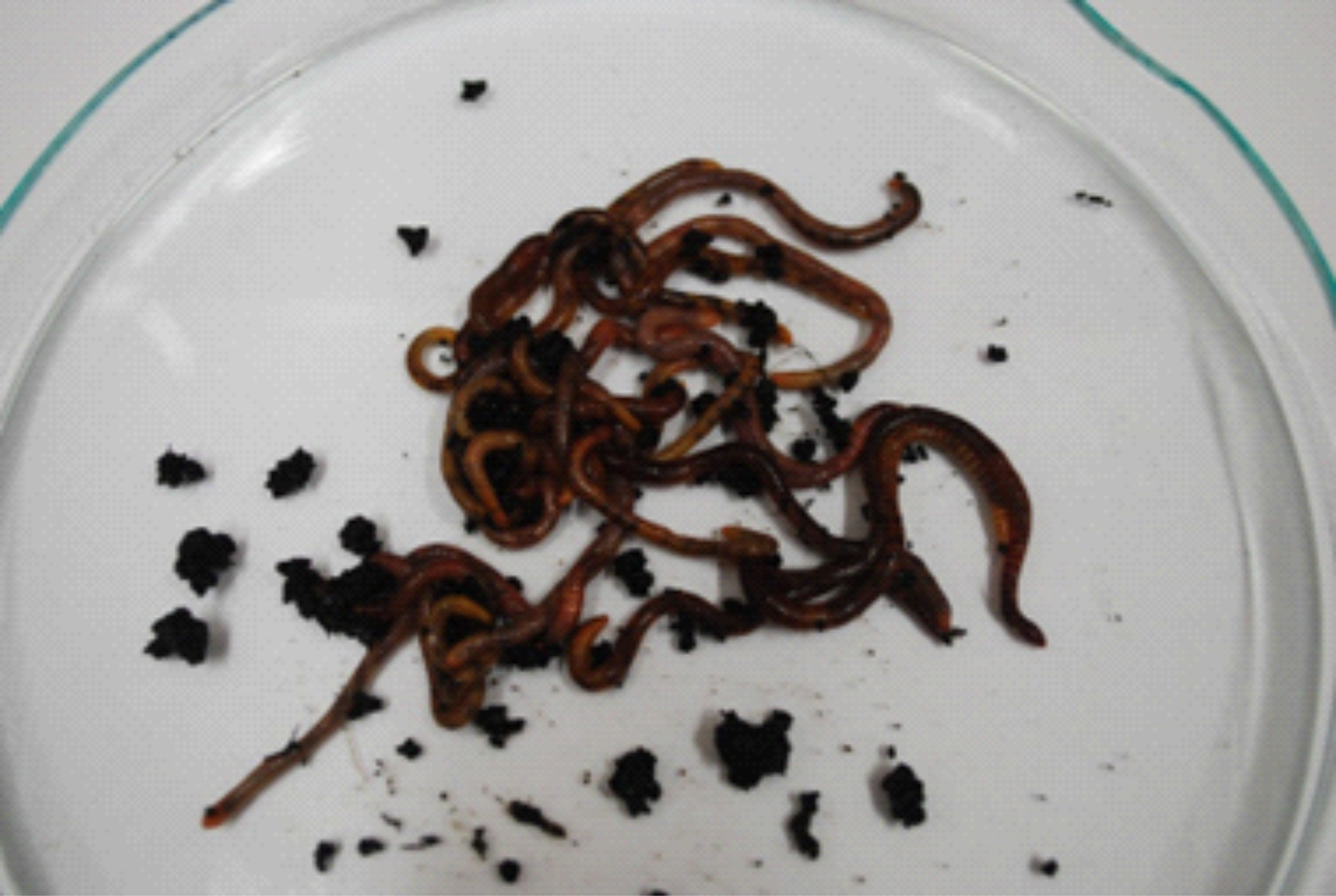

Supplement: Supplementary file 4 [file Image1.TIF]

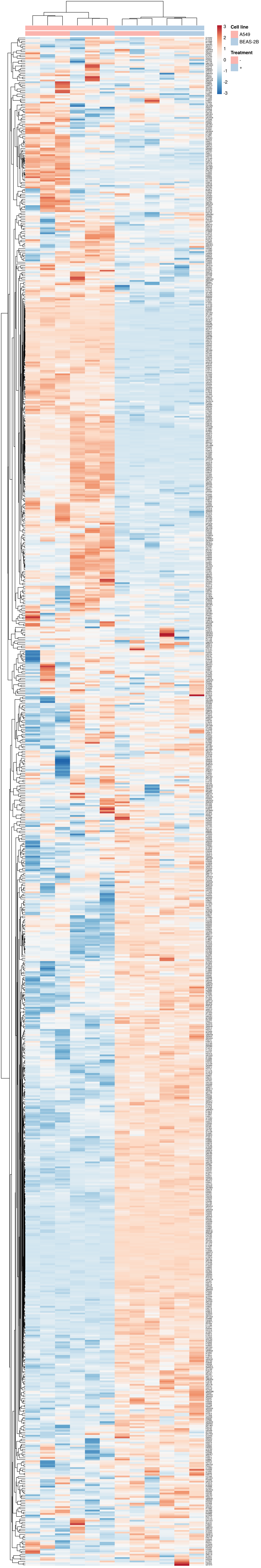

Supplement: Supplementary file 5 [file DataSheet1.PDF]
